# *Mycobacterium tuberculosis* Virulent Factor ESAT-6 Drives Macrophage Differentiation Toward the Pro-inflammatory M1 Phenotype and Subsequently Switches It to the Anti-inflammatory M2 Phenotype

**DOI:** 10.3389/fcimb.2018.00327

**Published:** 2018-09-18

**Authors:** Amira Refai, Sami Gritli, Mohamed-Ridha Barbouche, Makram Essafi

**Affiliations:** ^1^Laboratory of Transmission, Control and Immunobiology of Infections, Pasteur Institute of Tunis, Tunis, Tunisia; ^2^Department of Developmental Biology, Harvard School of Dental Medicine and Harvard Medical School, Boston, MA, United States

**Keywords:** tuberculosis, *M. tuberculosis*, ESAT-6, macrophages, polarization

## Abstract

Tuberculosis, a human infectious disease caused by *Mycobacterium tuberculosis* (*M.tb*), is still a major cause of morbidity and mortality worldwide. The success of *M.tb* as a pathogen relies mainly on its ability to divert the host innate immune responses. One way by which *M.tb* maintains a persistent infection in a “silent” granuloma is to inhibit inflammation and induce an immunoregulatory phenotype in host macrophages (MΦs). However, *M.tb* effectors governing the switch of MΦs from the pro-inflammatory M1 to the anti-inflammatory M2 phenotype remain to be determined. The *E*arly *S*ecreted *A*ntigenic *T*arget 6 kDa or ESAT-6, has been implicated in the virulence and pathogenesis of tuberculosis. Here, we investigated roles of ESAT-6 in MΦ differentiation and polarization. We found that treatment of human monocytes with ESAT-6 did not interfere with differentiation of M1 MΦs. However, ESAT-6 promoted differentiation of M0 and M2 MΦs toward the M1 phenotype, as indicated by secretion of pro-inflammatory cytokines IL-6, IL-12, and TNF-α, and induction of a typical M1 transcriptional signature. Interestingly, we found that ESAT-6 switched terminal full activation of M1 polarized MΦs to the M2 phenotype. Indeed, in the pro-inflammatory M1 MΦs, ESAT-6 was able to inhibit IL-12 and TNF-α secretion and stimulate that of IL-10. Moreover, gene expression profiling of these cells showed that ESAT-6 induced downregulation of M1 MΦ cell surface molecules *CD80* and *CD86*, transcription factors *IRF5* and *c-MAF*, cytokines *IL-12, IL-10*, and *IL-6*, as well as chemokines *CXCL10* and *CXCL1*. Overall, our findings suggest ESAT-6 as being one of the effectors used by *M.tb* to induce the pro-inflammatory M1 phenotype at the primo-infection; a prerequisite step to promote granuloma formation and subsequently drive the phenotype switch of MΦ polarization from M1 to M2 at a later stage of the infection. Our study improves current knowledge regarding mechanisms of virulence of *M.tb* and may be helpful to develop novel tools targeting ESAT-6 for a better and more efficient treatment of tuberculosis.

## Introduction

Tuberculosis, due to infection with *M.tb*, is a leading infectious disease and one of the major causes of death worldwide. Since 1993, the World Health Organization (WHO) declared tuberculosis as being a global public health emergency and intensified its efforts to control this severe airborne disease. Indeed, every year approximately 10 million new tuberculosis cases and 2 million deaths are reported around the globe (WHO, 2017).

In pulmonary tuberculosis for instance, *M.tb* infects the first immunological barrier, namely alveolar MΦs (Cambier et al., 2014; Orme, 2014). MΦs are versatile cells with a high degree of plasticity, thus exhibiting a considerable phenotypic diversity that confers them the ability to efficiently respond to environmental cues (Wang et al., 2014; Sica et al., 2015). MΦs play crucial roles in tuberculosis not only as a reservoir for *M.tb* intracellular growth, but also as important mycobacterial antigen-presenting cells (Cambier et al., 2014; Orme, 2014). Recently, the concept of MΦ activation and polarization has emerged (Sica and Mantovani, 2012; Martinez and Gordon, 2014; Murray et al., 2014; Wang et al., 2014; Sica et al., 2015; Murray, 2017). Two well established polarized phenotypes were recognized based on MΦ gene expression induced in response to pathogen and cytokine stimulation (Sica and Mantovani, 2012; Martinez and Gordon, 2014; Murray et al., 2014; Wang et al., 2014; Sica et al., 2015; Murray, 2017). MΦs were subsequently classified into the “classically activated M1” MΦ phenotype and the “alternatively activated M2” MΦ phenotype (Sica and Mantovani, 2012; Martinez and Gordon, 2014; Murray et al., 2014; Wang et al., 2014; Sica et al., 2015; Murray, 2017). M1 and M2 MΦ responses describe the opposing activities of killing and repairing, respectively. Activated M1 MΦs are considered potent effector cells of the innate immune response, which kill microorganisms and cancer cells and promote Th1 immune responses (Martinez and Gordon, 2014; Murray et al., 2014; Wang et al., 2014; Sica et al., 2015; Murray, 2017). Differentiation of monocytes into M1 MΦs occurs in presence of GM-CSF, while their full activation is obtained upon stimulation by IFN-γ alone or in conjunction with microbial products, such as lipopolysaccharide (LPS) or pro-inflammatory cytokines, such as TNF-α (Martinez and Gordon, 2014; Murray et al., 2014; Wang et al., 2014; Sica et al., 2015; Murray, 2017). Typical characteristics of M1 MΦs include high antigen presentation, high production of pro-inflammatory cytokines IL-12, IL-1β, IL-23, IL-6, and TNF-α, as well as high production of nitric oxide and reactive oxygen intermediates. M1 MΦ signature also involves upregulation of cyclooxygenase 2 (COX2) and transcription factor IRF5, expression of costimulatory molecules CD80 and CD86 and production of chemokines CXCL9, CXCL10, and CXCL11 (Mantovani et al., 2004; Benoit et al., 2008; Murray et al., 2014; Murray, 2017). By contrast, alternatively activated M2 MΦs are defined by low production of pro-inflammatory cytokines IL-12 and IL-23 and high production of the anti-inflammatory cytokine IL-10 (Martinez and Gordon, 2014; Murray et al., 2014; Wang et al., 2014; Sica et al., 2015; Murray, 2017). These cells show more phagocytic activity, higher expression of mannose and galactose receptors and scavenging molecules, as well as higher production of ornithine and polyamines (Mantovani et al., 2004; Sica and Mantovani, 2012). In general, M2 MΦs are poorly microbicidal, damp inflammation, have immunoregulatory functions and participate in polarized Th2 immune responses. The M2 polarized phenotype represents a generic name for several subsets of activated MΦs other than the classically M1 MΦs (Mantovani et al., 2004; Sica and Mantovani, 2012; Martinez and Gordon, 2014; Murray et al., 2014; Wang et al., 2014; Sica et al., 2015; Murray, 2017). Differentiation of monocytes into M2 MΦs is induced by M-CSF and their activation is obtained with IL-4, IL-13, immune complexes, IL-10, or glucocorticoids (Gordon and Martinez, 2010; Martinez and Gordon, 2014; Murray et al., 2014; Wang et al., 2014; Sica et al., 2015; Murray, 2017). M2 MΦs are characterized by upregulation of genes encoding *c-MAF, IRF4, TGF-*β, *IL-6, CCL1, CCL4, CCL13, CCL18, CXCL1, CXCL2*, and *CXCL3* (Mantovani et al., 2004; Murray et al., 2014; Murray, 2017). It has been shown that M1 and M2 polarized MΦ phenotypes can be reversible states, both *in vitro* and *in vivo* (Gratchev et al., 2006; Marino et al., 2015). A switch between pro- and anti-inflammatory cytokine profiles is also seen during this phenotypic reversibility (Muller and Tjardes, 2003).

Several pathogens developed strategies to interfere with the M1-associated killing phenotype and were shown to drive cell phenotypic switch to the M2 polarized phenotype (Sica et al., 2015). This is also the case of *M.tb* that was suggested to use such strategy to manipulate the infection fate through its interaction with MΦs. Indeed, it has recently been proposed that the mechanism of MΦ polarization plays a key role in granuloma formation and disease progression (Flynn et al., 2011; Lugo-Villarino et al., 2011, 2013; Sica et al., 2015). The early phase of *M.tb* infection is marked by the presence of M1 MΦs and a Th1 immune response, which is typically the response associated with protection against the disease (Benoit et al., 2008; Day et al., 2009). As the infection progresses in patients with tuberculosis, IFN-γ amounts in bronchoalveolar lavage and iNOS expression by MΦs decrease, whereas expression of the arginase 1 increases, indicating a re-polarization of MΦs toward the M2 phenotype (Ehrt et al., 2001; Redente et al., 2010; Lugo-Villarino et al., 2011, 2013). At a later stage of the infection, the observed transition is associated with high levels of Th2 cytokines, such as IL-4, IL-10, TGF-β, and IL-13, which correlates with disease severity (Ly et al., 2007; Raju et al., 2008; Almeida et al., 2009; Redente et al., 2010). However, little is known about the virulent factors and mechanisms involved in MΦ polarization.

One of the key determinants of mycobacterial pathogenicity is the region of difference 1, which is present in the genome of virulent *M.tb* but absent in the genome of the attenuated vaccine strain *Mycobacterium bovis* Bacillus Calmette-Guérin (BCG) (Samten et al., 2011). The chromosomal locus of region of difference 1 encodes the major secreted virulent factor, namely *ESAT-6* (Samten et al., 2011). Gains or losses of mycobacterial virulence are closely linked to the ability of mycobacteria to secrete ESAT-6 (Yu and Xie, 2012; Peng and Sun, 2016). Many crucial functions of *M.tb* are ascribed to ESAT-6, including membrane lytic activity that causes alveolar epithelial cell and MΦ damage (Ma et al., 2015) and destabilization of the phagolysosome allowing *M.tb* to translocate from the phagosome to the cytosol (Houben et al., 2012; Simeone et al., 2012). Cytosolic ESAT-6 translocation has multiple consequences on mycobacterial infection that essentially aims at interfering with the host cell defense (Peng and Sun, 2016). Manipulation of several intracellular signaling pathways in MΦs and T cells by ESAT-6 has also been reported (Yu and Xie, 2012). Secreted ESAT-6 was shown to induce MΦ apoptosis, which is partly related to upregulation of caspase activity (Derrick and Morris, 2007), cleavage of BAT3 and proteasomal degradation (Grover and Izzo, 2012) and induction of miR-155 expression through TLR2/NF-κB activation (Yang et al., 2015), as well as accumulation of reactive oxidative species, eventually leading to endoplasmic reticulum stress-induced apoptosis (Choi et al., 2010). ESAT-6 was also shown to reduce efficient MΦ antigen presentation through induction of class II MHC expression by inhibiting class II MHC transactivator expression and regulation of chromatin remodeling (Kumar et al., 2012) and to inhibit cell surface expression of class I MHC β2 microglobin complexes by sequestration of β2 microglobin (Sreejit et al., 2014). Ectopic expression of ESAT-6 and CFP-10 fusion protein can regulate autophagosome formation via modulation of expression of autophagy-related genes (Yu and Xie, 2012; Zhang et al., 2012). Additional reports showed that ESAT-6 is able to block autophagosome-lysosome fusion in a mammalian target of rapamycin-dependent manner and to repress autophagy flux (Yu and Xie, 2012; Dong et al., 2016). Other reports showed ESAT-6 as being the key mycobacterial effector responsible for foamy macrophage (FM) differentiation (Singh et al., 2012). Exposure of mice to ESAT-6 induces localized inflammatory cell aggregates with characteristics of an early granuloma (Boggaram et al., 2013). ESAT-6 was also shown to stimulate MMP9 production by host epithelial cells, which enhances recruitment of additional MΦs into the granuloma (Van den Steen et al., 2008; Volkman et al., 2010). Furthermore, ESAT-6 was shown to interfere with TLR signaling by its direct binding to TLR2, thus preventing the interaction between MyD88 and IRAK4; a crucial step in pro-inflammatory signalosome MyD88-IRAK-IKK formation (Pathak et al., 2007). This effect blocks NF-κB and interferon regulatory factor activation and inhibits LPS-induced production of IL-12p40, IL-6, and TNF-α by murine MΦs, which are hallmarks of the anti-inflammatory response (Pathak et al., 2007).

Collectively, the above-mentioned data underscore the importance of ESAT-6 as a mediator implicated in the modulation of the inflammatory response. It is therefore interesting to test whether ESAT-6 is involved in *M.tb*-induced MΦ polarization.

In the present study, we investigated the potential effects of ESAT-6 on MΦ differentiation, activation, and polarization. We found that ESAT-6 did not interfere with differentiation of M1 MΦs, but promoted differentiation of M0 and M2 MΦs toward the M1 phenotype. Interestingly, we found that ESAT-6 modulated expression of pro-inflammatory markers of fully activated M1 MΦs. Our results suggest ESAT-6 as being a key virulent effector used by *M.tb*, to drive host MΦ differentiation and activation as a mechanism to subvert the immune response in order to maintain a persistent infection.

## Materials and methods

### Expression and purification of ESAT-6

Expression and purification of recombinant ESAT-6 were achieved in non-denaturing conditions and without detergent treatment as previously described by our group (Refai et al., 2015). In these conditions, ESAT-6 was generated as a dimeric complex that mimics the physiologic heterodimer ESAT-6/CFP10. Briefly, *E. coli* BL21 were transformed with a construct encoding *ESAT-6* and *pET23b-Rv3875* (*esxA*). Transformed cells were grown in Luria Broth medium (Sigma-Aldrich, St. Louis, MO) containing 100 μg/mL ampicillin (Sigma-Aldrich, St. Louis, MO). Expression of ESAT-6 was induced for 5 h at 37°C using 0.25 mM IPTG (Euromedex, Souffelweyersheim, France). Cells were collected and sonicated in lysis buffer containing 20 mM Tris-HCl, pH 8, 500 mM NaCl, 5 mM imidazole, EDTA-free protease inhibitor cocktail (Roche LifeScience, Mannheim, Germany), 1.8 mg/mL DNase 1 (Roche LifeScience, Mannheim, Germany) and 200 μg/mL lysozyme (Sigma-Aldrich, St. Louis, MO). The recombinant His-tagged ESAT-6 was then purified on a nickel column (Bio-Rad, Hercules, CA). PD-10 column was used to exchange the elution buffer by 1X PBS (ThermoFisher Scientific, Cambridge, MA). Protein quantification was achieved using Bicinchoninic Acid Protein Assay (Sigma-Aldrich, St. Louis, MO). Endotoxin level in the ESAT-6 preparation was measured using Pierce LAL Chromogenic Endotoxin Quantitation Kit (ThermoFisher Scientific, Cambridge, MA) following the manufacturer's recommendations and was found to be as low as 0.54 EU/mg of ESAT-6 protein.

### Generation and polarization of human monocyte-derived macrophages

#### Human monocyte isolation and purification

Informed consent was obtained from subjects enrolled in the study and the experimental protocol was approved by the Institutional Review Board of the Pasteur Institute of Tunis. Peripheral blood human mononuclear cells (PBMCs) were collected from whole fresh blood of three healthy volunteers as previously described by our group (Haoues et al., 2014). These donors were negative for any recent infection and had no history of tuberculosis. Briefly, PBMCs were separated by density gradient using Ficoll-Paque TM PLUS (GE Healthcare, Boston, MA) according to the manufacturer's instructions. Cells were then washed, re-suspended in RPMI 1640 (ThermoFisher Scientific, Cambridge, MA) containing 2 mM L-glutamine, 100 U/mL penicillin, 100 μg/mL streptomycin (all from ThermoFisher Scientific, Cambridge, MA) and 10% of heat-inactivated human AB serum. Cells were seeded in T75 flasks (BD Biosciences, San Jose, CA) at a density of 10^6^ cells/mL. Monocytes were isolated from PBMCs by adherence on gelatin-coated plates (Sigma-Aldrich, St. Louis MO). Non-adherent cells were removed by gentle pipette aspiration after 2 h of incubation at 37°C in a humidified atmosphere containing 5% CO_2_. Monocytes were then detached from the flasks by a 15 min incubation in medium containing 50% RPMI 1640, 50% 1X PBS, 5mM EDTA and 5% fetal bovine serum (FBS) at 37°C. Collected cells were counted and monocyte purity was evaluated by flow cytometry using a Canto II fluorescence-activated cell sorter (BD Biosciences, San Jose, CA) and FITC-coupled anti-human CD14, CD3, and CD19 antibodies (BD Biosciences, San Jose, CA). Monocyte purity was greater than 90%.

#### Differentiation of monocytes into various macrophage subpopulations

To differentiate monocytes into MΦs, cells were cultured for 7 days at 37°C in a humidified atmosphere containing 5% CO_2_. Non-polarized M0 MΦs (non-activated) were obtained by culturing monocytes in RPMI 1640 containing 2 mM L-glutamine, 100 U/mL penicillin, 100 μg/mL streptomycin, 10% FBS (complete medium) (all from ThermoFisher Scientific, Cambridge, MA), supplemented with 10% heat-inactivated human AB serum. Polarization of monocytes toward M1 MΦs was obtained by incubating cells in complete medium supplemented with 100 ng/mL of human recombinant GM-CSF (ImmunoTools, Fryesoythe, Germany), while polarization toward M2 MΦs was induced by adding 10 ng/mL of human recombinant M-CSF (ImmunoTools, Fryesoythe, Germany) to complete medium. Upon cell differentiation, full activation of polarized MΦs was obtained using activating molecules for an additional 24 h. After 6 days of culture, activated M1 (M1A) MΦs were indeed obtained by stimulation of M1 MΦs with 10 ng/mL LPS (Sigma-Aldrich, St. Louis, MO) and 20 ng/mL IFN-γ (ImmunoTools, Fryesoythe, Germany), whereas M2 activated (M2A) MΦs were obtained after induction of M2 MΦs with 20 ng/mL IL-4 (ImmunoTools, Fryesoythe, Germany) and 20 ng/mL IL-13 (ImmunoTools, Fryesoythe, Germany). After 24 h of culture, supernatants were collected and stored at −20°C for cytokine assays, while cells were lysed in TRIzol (Gibco BRL, Life Technologies, Rockville, MD) and stored at −80°C until use. Assessment of effects of ESAT-6 on MΦ differentiation and/or polarization was carried out after adding 10 μg/mL of the appropriate recombinant protein to culture media. Except for M1A activated macrophages, all cells were pretreated with 10 μg/mL of polymyxin B (Sigma-Aldrich, St. Louis, MO) for 30 min to avoid any potential effect of *E. Coli*-derived LPS.

#### ELISA cytokine assays

Human IL-12, TNF-α, IL-6, and IL-10 ELISA Set BD OptEIA™ kits (BD Biosciences, San Jose, CA) were used to quantify the secreted cytokines in cell culture supernatants, according to the manufacturer's instructions.

#### Reverse transcription

Total RNA was extracted from cells using RNeasy Mini Kit (Qiagen, Hilden, Germany) according to manufacturer's instructions and treated with DNase-I on the column. Briefly, cells were homogenized using an appropriate volume of TRIzol. Chloroform (0.2 /1mL TRIzol) was then added to separate the aqueous phase from the organic one. Total RNA was then precipitated from the aqueous phase. After several washings, RNA was treated with DNase-I, eluted and stored in RNase-free water. RNA sample concentration and quality were determined using ThermoFisher Scientific Nanodrop 1000 (Cambridge, MA). RNA was then reverse-transcribed into cDNA in a final volume of 20 μL using Applied Biosystems high capacity cDNA reverse transcription kit (Foster City, CA) following the manufacturer's instructions. For cDNA synthesis, reactions were carried out at 25°C for 10 min, 42°C for 120 min and 95°C for 5 min. cDNA products were stored at −20°C until use.

#### Real-time quantitative PCR (RT-qPCR)

Specific cDNA levels were quantified by RT-qPCR using the Platinum® SYBR Green qPCR Supermix-UDG w/ROX (Life Technologies, Carlsbad, CA) and run in a 7900HT Fast Real-time System (Applied Biosystems, Foster City, CA). cDNA was amplified in a final volume of 25 μL containing 10 ng of cDNA template, 12.5 μL of 2X QuantiTect SYBR Green PCR Master Mix and M1/M2 specific primers at a final concentration of 200 nM. After an incubation at 50°C for 20 min, amplification comprised 40 cycles as follows: 95°C for 15 s, 60°C for 15s and a final cycle at 60°C for 10 min. Primers for qPCR are as follows: *CD80*: F 5′-CAGGGAACATCACCATCCAA-3′, R 5′-CAGCGTTGCCACTTCTTTCA-3′, *CD86*: F 5′-AGCGGCCTCGCAACTCTTAT-3′, R 5′-AAAACACGCTGGGCTTCATC-3′,

*COX2*: F 5′-CTCCTGTGCCTGATGATTGC-3′, R 5′-TGGGGATCAGGGATGAACTT-3′,

*IRF5*: F 5′-TCTGCTTTGGGGAAGAATGG-3′, R 5′-TGACCAAGATAGCTCCCCTGA-3′, *c-MAF*: F 5′-AGTCCCCTGGCCATGGAATA-3′, R 5′-GCGGGTTTGTGTGTGTGTAG-3′, *CXCL10*: F 5′-GAACCTCCAGTCTCAGCACC-3′, R 5′-GCGGGTTTGTGTGTGTGTAG-3′, *CCL1*: F 5′-AGCCAGACCAGAAGACATGC-3′, R 5′-GGTAGTTTCGGGGACAGGTG-3′, *CCL18*: F 5′-CTTGTCCTCGTCTGCACCAT-3′, R 5′-AGTTAGTTAATGTGGCTGGGCA-3′, *IL-12p40*: F 5′-GTGGATGCCGTTCACAAGCTC-3′, R 5′-CCTCCACCTGCCGAGAATTC-3′, *IL-1*β: F 5′-CAGGCTGCTCTGGGATTCTC-3′, R 5′-TCCACATTCAGCACAGGACTC-3′, *IL-23*: F 5′-TGAACAGAGAGAATCAGGCTC-3′, R 5′-GGGCCATGGAGATCTGAGTG-3′, *IL-6*: F 5′-AATCATCACTGGTCTTTTGGAG-3′, R 5′-GGTTATTGCATCTAGATTCTTTGC-3′, *IL-10*: F 5′-AACAAGAGCAAGGCCGTGGA-3′, R 5′-GAAGATGTCAAACTCACTCATGGC-3′. Controls included: *GAPDH*: F 5′- CCATCAATGACCCCTTCATTG-3′, R 5′-CTTGACGGTGCCATGGAATT-3′, *HPRT*: F 5′-CCCTGGCGTCGTGATTAG-3′, R 5′-ATGGCCTCCCATCTCCTT-3′, *ubiquitin*: F 5′-CCGACCACAGTGGCTATGC-3′, R 5′-CCTCTTTTAATATCTCCAGGCTTGA-3′ and β*-actin*: F 5′-CACGGCATCGTCACCAACT-3′, R 5′-AGCCACACGCAGCTCATTG-3′. Specificity of PCR reactions was verified by melting curve analysis of each amplified product using the Applied Biosystems 7500 Fast Real-Time PCR System Software. Each real-time PCR reaction was carried out in triplicate. Relative gene expression was calculated using the target threshold cycle value (Ct) and the 2^−ΔΔ^Ct method.

### Statistical analyses

All statistical analyses were done using GraphPad Prism 6 (Graph Pad Software, La Jolla, CA). Data were presented as means ± *SD*. The unpaired Student's *t*-test was used for simple comparison and the ANOVA for multiple comparisons. Significance is indicated by a symbol. Differences with a *p*-value < 0.05 were considered statistically significant. In order to further verify the accuracy of the data obtained using these statistical tests, we also applied the one-tailed version of the Mann Whitney test, which is more suited for small-sized samples (Salkind, 2007; Morgan, 2017), as it is the case in our study.

## Results

### ESAT-6 does not interfere with M1 differentiation but directs M0 and M2 differentiation toward the M1 phenotype

**Figure 1:**
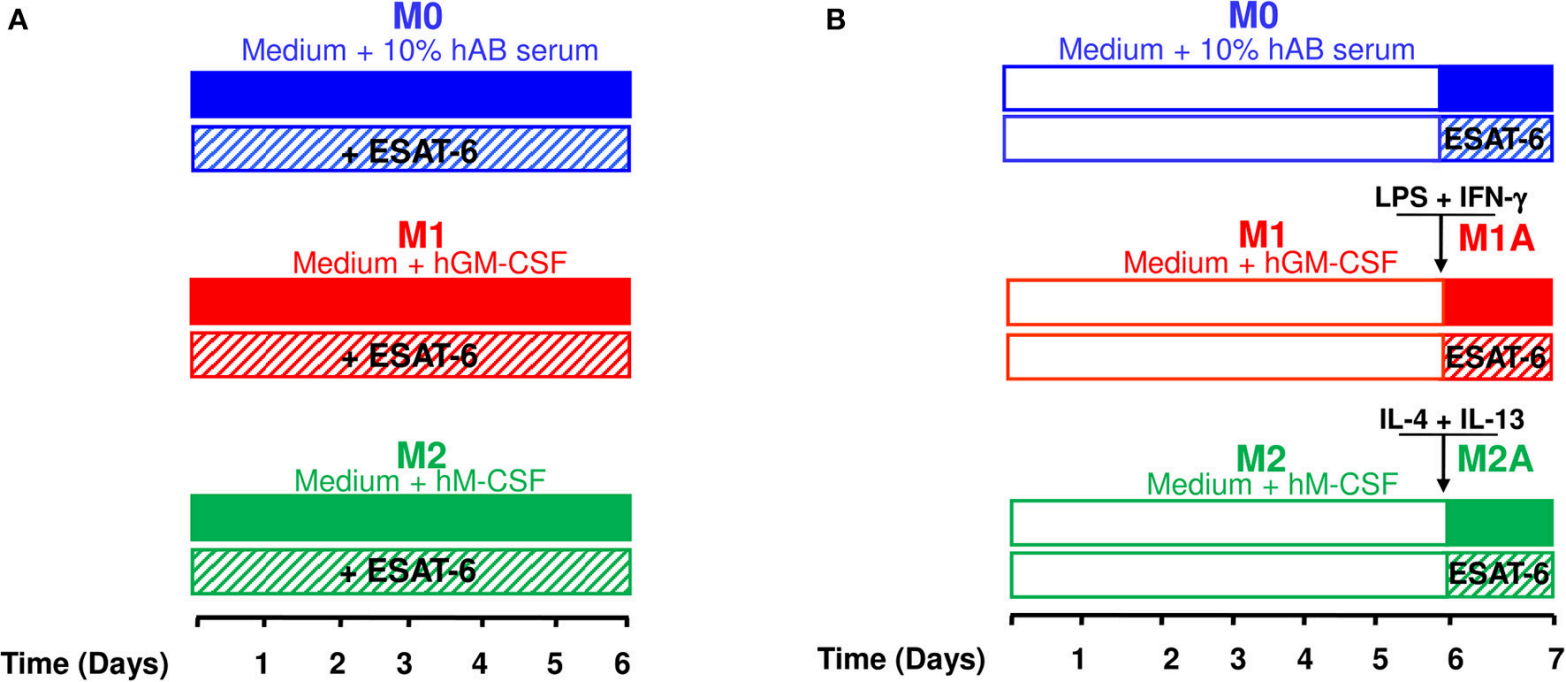
Experimental procedures used to generate different macrophage subpopulations. The experimental procedures carried out to assess effects of ESAT-6 on MΦ differentiation, polarization and activation were based on the recommended protocol guidelines (Murray et al., 2014). **(A)** Human-derived monocytes, from three different donors, were incubated in medium containing human AB serum for 6 days to obtain non-polarized M0 MΦs. M1 and M2 MΦs were obtained by adding 100 ng/mL GM-CSF and 10 ng/mL M-CSF, respectively. Effects of ESAT-6 on MΦ differentiation were assessed by adding 10μg/mL of the recombinant protein. **(B)** To generate fully activated (M1A and M2A) MΦs, cells were treated as in **A** for 6 days before adding for 24 h the activation cocktail (10 ng/mL LPS and 20 ng/mL IFN-γ for M1A MΦs; 20 ng/mL IL-4 and 20 ng/mL IL-13 for M2A MΦs). Effects of ESAT-6 on fully activated MΦs were evaluated by adding 10 μg/mL of the recombinant protein to the activation cocktail at day 6.

**Figure 2:**
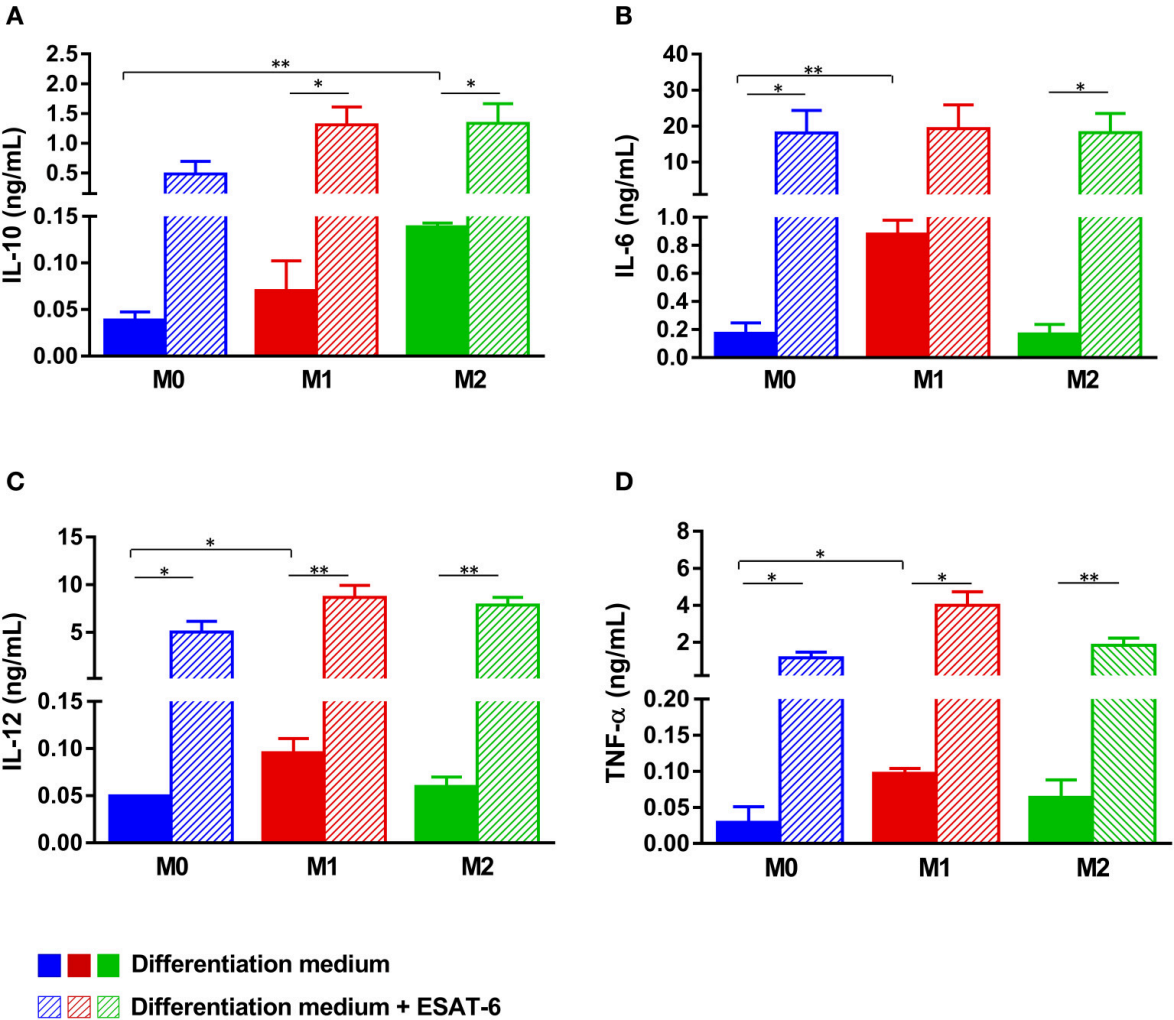
ESAT-6 enhances the pro-inflammatory cytokine secretion by non-polarized M0 and M2 macrophages. Monocytes were differentiated into (i) M0 MΦs by incubation in medium containing human AB serum, (ii) M1 MΦs in medium containing 100 ng/mL GM-CSF and (iii) M2 MΦs in medium containing 10 ng/mL M-CSF. Effects of ESAT-6 were assessed by adding 10 μg/mL of the recombinant protein. Six days later, secretion of IL-10 **(A)**, IL-6 **(B)**, IL-12 **(C)**, and TNF-α **(D)** was quantified by ELISA. Data are presented as the mean ± *SD* of 3 separate experiments (i.e., 3 different donors), each one carried out in triplicate. Asterisks indicate statistical significance (*p ≤ 0.05, **p ≤ 0.01).

ESAT-6 is always secreted by *M.tb* as a protein complex ESAT-6/CFP-10 stabilized by hydrophobic interactions (Yu and Xie, 2012; Peng and Sun, 2016). We previously reported that in the absence of its partner CFP-10, recombinant ESAT-6 adopts a dimeric conformation whose biological functions mimic those of the physiologic ESAT-6/CFP-10 complex (Refai et al., 2015). We then purified such form in order to assess its effects on MΦ differentiation. Human monocytes were treated as detailed in material and methods to generate M0, M1, or M2 MΦs either in the presence or absence of recombinant ESAT-6 (Figure 1A). When ESAT-6 is not present in medium, we found that M0 MΦs secreted low amounts of IL-10, IL-6, IL-12, and TNF-α, while GM-CSF- or M-CSF-induced monocyte differentiation into M1 or M2 MΦs, respectively, yielded moderate levels of the studied cytokines (Figures 2A–D). As expected, the cytokine profile showed that M1 MΦs secreted significantly higher amounts of IL-6 (*p* = 0.005, Figure 2B), IL-12 (*p* = 0.046, Figure 2C) and TNF-α (*p* = 0.048, Figure 2D) than M0 MΦs, whereas almost no pro-inflammatory cytokines were secreted by M2 MΦs (Figures 2B–D). However, the latter ones secreted significantly higher levels of IL-10 than M0 MΦs (*p* = 0.004, Figure 2A). These results suggest that the protocols we used herein successfully allowed the generation of distinct MΦ subpopulations, namely non-polarized M0, pro-inflammatory M1 and anti-inflammatory M2 phenotypes. Addition of ESAT-6 to medium during M0 MΦ differentiation induced significant levels of IL-6 (*p* = 0.022, Figure 2B), IL-12 (*p* = 0.021, Figure 2C) and TNF-α (*p* = 0.011, Figure 2D), while induction of IL-10 was not that significant (*p* = 0.09, Figure 2A). This suggests that ESAT-6 induced non-polarized M0 MΦs to switch to the M1 phenotype. Treatment of monocytes with ESAT-6 during their differentiation into either M1 or M2 MΦs induced a significant secretion of the 4 studied cytokines, though without driving them to differentiate into a distinct polarization phenotype, as we rather found a “mixed M1/M2” MΦ profile. Nevertheless, we found that induction of IL-6 and TNF-α by ESAT-6 was statistically more significant in M2-differentiated MΦs (*p* = 0.014, Figure 2B; *p* = 0.0054, Figure 2D) than in M1-differentiated MΦs (*p* = 0.06, Figure 2B; *p* = 0.016, Figure 2D). This again suggests that during monocyte differentiation, ESAT-6 pushes the balance toward a pro-inflammatory MΦ phenotype.

**Figure 3:**
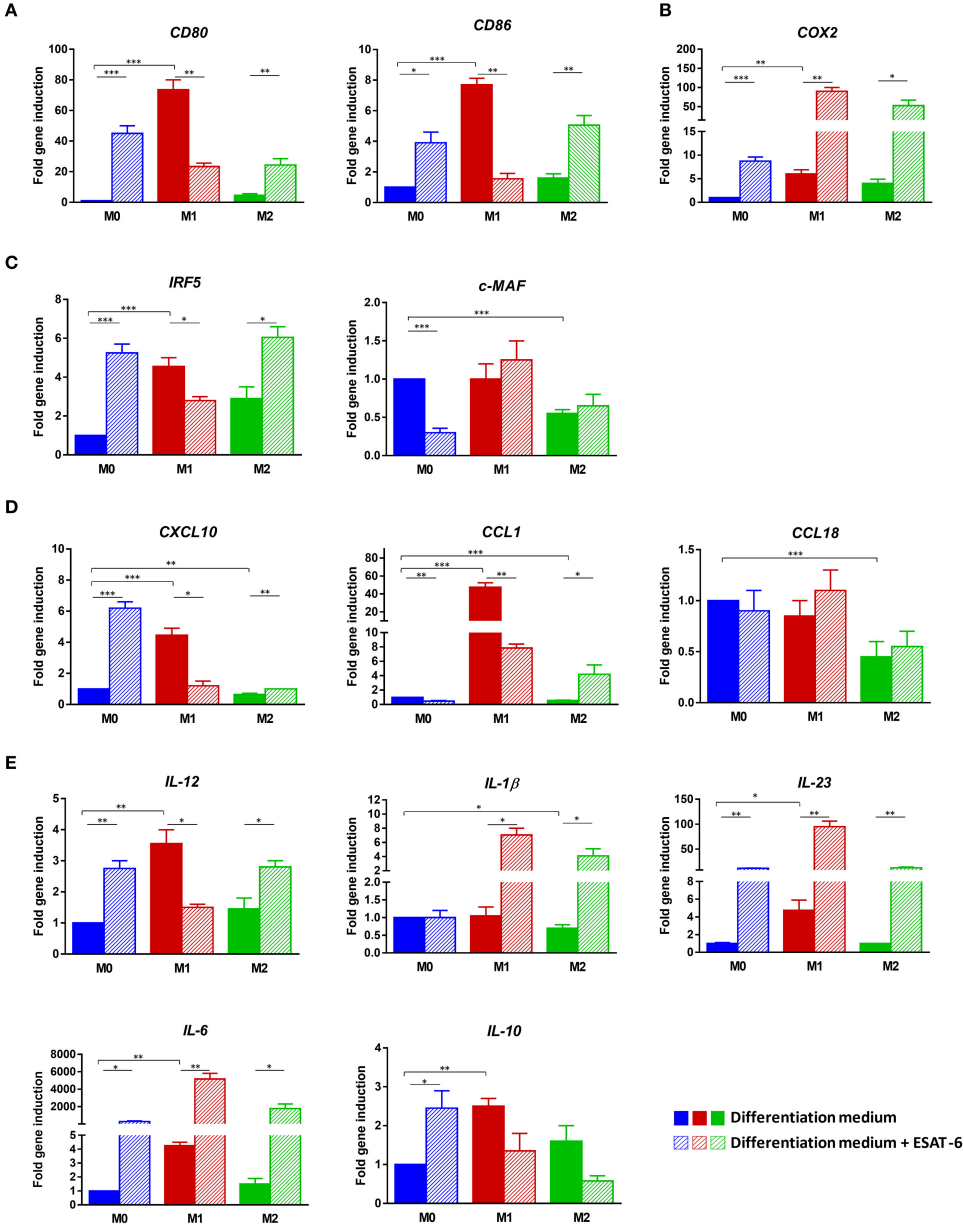
ESAT-6 directs M0 and M2 macrophage differentiation toward the M1 phenotype. Non-polarized M0 MΦs, pro-inflammatory M1 MΦs and anti-inflammatory M2 MΦs were differentiated from human purified monocytes as above-indicated. Cells were either treated or not treated with 10 μg/mL ESAT-6. After 6 days of MΦ differentiation, mRNAs were extracted and expression levels of different MΦ markers were assessed by RT-qPCR. Fold changes in gene expression of **(A)** Cell surface molecules *CD80* and *CD86*, **(B)** *COX2*, **(C)** Transcription factors *IRF5* and *c-MAF*, **(D)** Chemokines *CXCL10, CCL1*, and *CCL18*, as well as **(E)** Cytokines *IL-12, IL-1*β, *IL-23, IL-6*, and *IL-10* are shown. Data are presented as the mean ± *SD* of 3 separate experiments (i.e., 3 different donors), each one carried out in triplicate.

In order to better characterize the observed profile during MΦ differentiation, we studied gene expression levels of M1 and M2 MΦ characteristic markers, using RT-qPCR in the 3 generated MΦ subpopulations M0, M1, and M2. Expression levels of *CD80, CD86, COX2, IRF5, c-MAF, CXCL10, CCL1, CCL18, IL-12, IL-1*β, *IL-23, IL-6*, and *IL-10* were assessed either in the presence or absence of ESAT-6. When ESAT-6 is not present in medium, the gene expression profile observed in M1-differentiated MΦs clearly confirmed a typical M1 signature (Figure 3), in agreement with the cytokine profile we described above. Indeed, we found a significant increase in two M1 MΦ markers, *CD80* and *CD86*, when compared to that seen in M0 MΦs (*p* < 0.001, Figure 3A). We also detected a significant induction of *COX2* (*p* < 0.01, Figure 3B), a striking increase in *IRF5* (*p* < 0.001, Figure 3C) and significant high expression levels of *CXCL10* (*p* < 0.001, Figure 3D), *IL-12, IL-23*, and *IL-6* (p < 0.01, Figure 3E). In M2-differentiated MΦs, we found no induction of *CXCL10* (Figure 3D), *IL-12, IL-23*, and *IL-6* (Figure 3E) and a much less significant increase in *CD80, CD86*, and *COX2* (Figure 3A) and *IRF5* (Figure 3B). Moreover, expression of *IL-1*β significantly decreased in M2 MΦs when compared to that seen in M0 MΦs (*p* = 0.014, Figure 3E). These data indicate that the protocols we used successfully allowed generation of the M2 phenotype. Addition of ESAT-6 to M0 MΦ differentiation medium significantly enhanced expression levels of M1 markers *CD80* (*p* = 0.0007), *CD86* (*p* = 0.014), and *COX2* (*p* = 0.0006), as shown in Figure 3A. We also observed a significant increase in expression levels of M1 marker *IRF5* (*p* = 0.0005, Figure 3C) and a significant decrease in M2 marker *c-MAF* (*p* = 0.0001, Figure 3C). This again indicates that ESAT-6 has directed differentiation of M0 MΦs toward the M1 phenotype. Such observation is further supported by the strong induction of M1 marker *CXCL10* (*p* = 0.0002, Figure 3D) and almost complete inhibition of M2 marker *CCL1* (*p* = 0.002, Figure 3D). Furthermore, we found that ESAT-6 led to a significant induction of expression levels of M1 cytokines *IL-12 (p* = 0.0013), *IL-23*, (*p* = 0.0023), and *IL-6* (*p* = 0.03) (Figure 3E). In M2-differentiated MΦs, ESAT-6 has also significantly induced expression of M1 markers *CD80* and *CD86* (*p* < 0.01, Figure 3A), *COX2* (*p* = 0.04, Figure 3B), *IRF5* (*p* = 0.03, Figure 3C), *CXCL10* (*p* = 0.007, Figure 3D), *IL-12, IL-1*β, and *IL-6* (*p* < 0.05, Figure 3E) and *IL-23* (*p* = 0.007, Figure 3E). These data suggest that during MΦ differentiation, ESAT-6 has also induced a phenotype switch from M2 to M1. However, ESAT-6 variably affected (either induction or inhibition) expression levels of the studied markers in differentiated M1 MΦs (Figures 3A–E).

### ESAT-6 modulates the pro-inflammatory cytokine secretion by fully activated M1 macrophages

**Figure 4:**
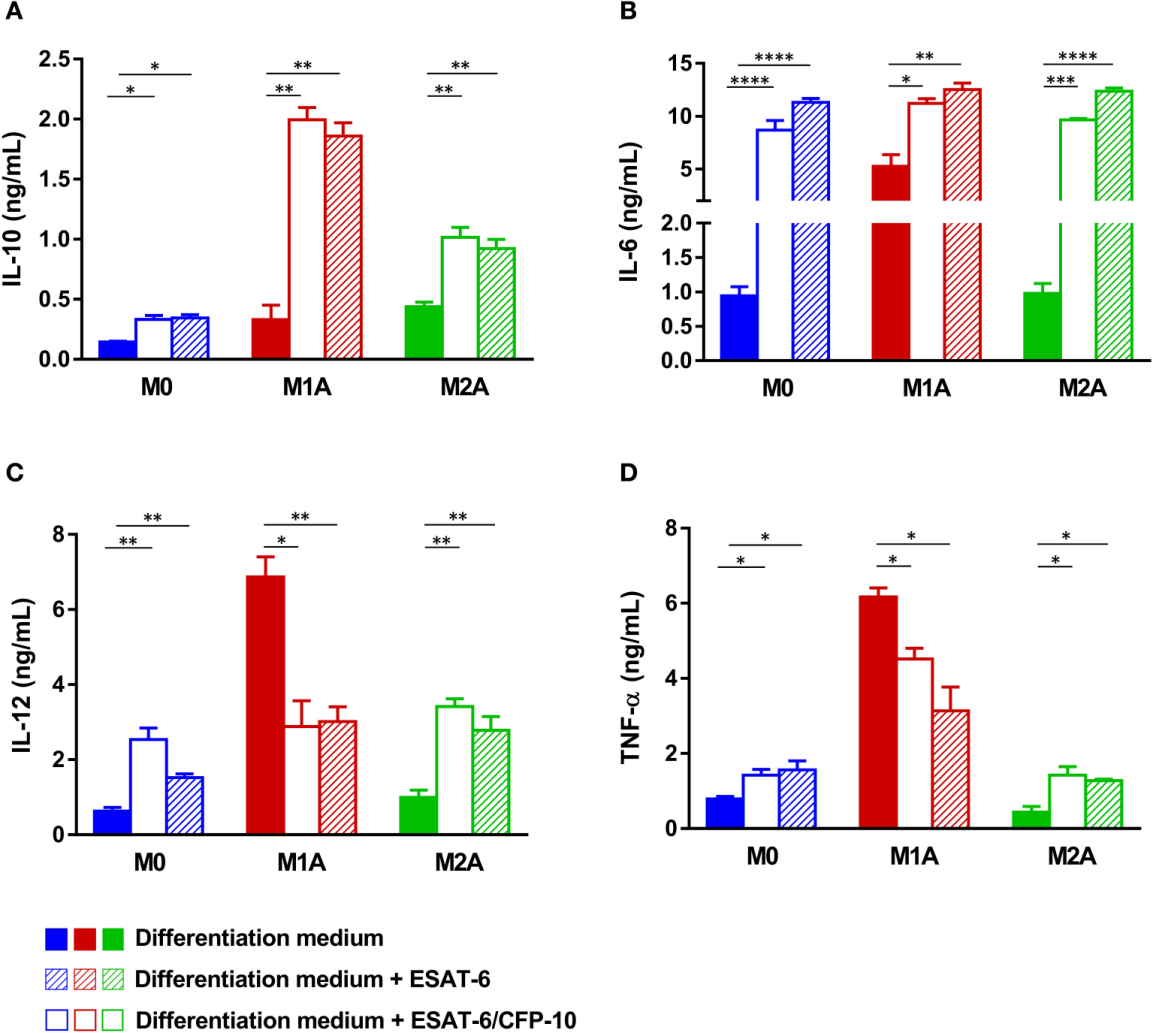
Both ESAT-6 and the physiologic ESAT-6/CFP10 complex inhibit secretion of M1A cytokines. After 6 days of MΦ differentiation as indicated in Figure 1, M1 MΦs were stimulated in presence or not of 10 μg/mL ESAT-6 in combination with 10 ng/mL LPS and 20 ng/mL IFN-γ to generate fully activated M1A MΦs. Fully activated M2A MΦs were generated by adding 20 ng/mL IL-4 and 20 ng/mL IL-13 to M2 differentiation medium, in presence or not of 10 μg/mL ESAT-6 as indicated in Figure 1. Assessment of the effects of the physiologic ESAT-6/CFP10 complex was similarly performed. After 24 h of incubation, supernatants were collected and secretion levels of **(A)** IL-10, **(B)** IL-6, **(C)** IL-12, and **(D)** TNF-α were assessed. Data are presented as the mean ± *SD* of 3 separate experiments (i.e. 3 different donors), each one carried out in triplicate (*p ≤ 0.05, **p ≤ 0.01, ***p ≤ 0.001).

A complete polarization and efficient full activation of MΦs into either a pro-inflammatory M1 (M1A) or anti-inflammatory M2 (M2A) phenotype can be achieved via stimulation of the differentiated cells by several factors, which could be pathogens, apoptotic or necrotic cells, chemokines, lipids, or cytokines present in the microenvironment (Sica and Mantovani, 2012; Martinez and Gordon, 2014; Wang et al., 2014; Sica et al., 2015; Murray, 2017). In order to assess effects of ESAT-6 on fully activated MΦs, we added the protein to M0, M1A, or M2A MΦs at day 6, as indicated in Figure 1B. Effects of ESAT-6 on cytokine secretion by M0 MΦs were similar to those seen in the above-mentioned differentiated MΦs (Figures 2A–D), except for IL-10, which was significantly induced when ESAT-6 was added at day 6 for an additional 24 h (*p* = 0.017, Figure 4A). As expected, M1A MΦs produced higher levels of IL-6, IL-12, and TNF-α than M1 MΦs. M1A MΦs also generated significantly higher levels of the above-mentioned 3 pro-inflammatory cytokines than M0 and M2A MΦs (Figures 4B–D), while M2A MΦs produced higher levels of IL-10 than M0 and M1A MΦs (Figure 4A). These data also indicate our success regarding *in vitro* full activation of the two MΦ subpopulations by the combination of LPS and IFN-γ in M1A MΦs and IL-4 and IL-13 in M2A MΦs. Treatment of M1A MΦs with ESAT-6 significantly inhibited secretion of IL-12 (p = 0.0054, Figure 4C) and TNF-α (*p* = 0.032, Figure 4D), while significantly induced that of IL-10 (*p* = 0.0042, Figure 4A). We found no significant effect of ESAT-6 on IL-6 secretion by M1A MΦs (Figure 4B). Our results suggest that ESAT-6 modulates the pro-inflammatory phenotype of M1A MΦs and directs it toward the anti-inflammatory M2 phenotype (Figures 4A–D).

To gain further insights into roles of ESAT-6 in MΦ polarization, we used a recombinant form of the physiologic heterodimer ESAT-6/CFP10 to treat the different MΦ subpopulations M0, M1A, and M2A. As expected and in agreement with our previous report (Refai et al., 2015), ESAT-6/CFP10 complex behaved similarly as the dimeric ESAT-6 by modulating M1A MΦ secretion levels; with a decrease in IL-12 (*p* = 0.018, Figure 4C) and TNF-α (*p* = 0.0108, Figure 4D) levels, but increase in IL-10 levels (*p* = 0.0024, Figure 4A). However, treatment of M2A and M0 MΦs either with ESAT-6 or with the ESAT-6/CFP10 complex induced secretion of all 4 studied cytokines without directing them to any distinct polarization phenotype (Figures 4A–D).

### ESAT-6 modulates expression of M1A macrophage markers

**Figure 5:**
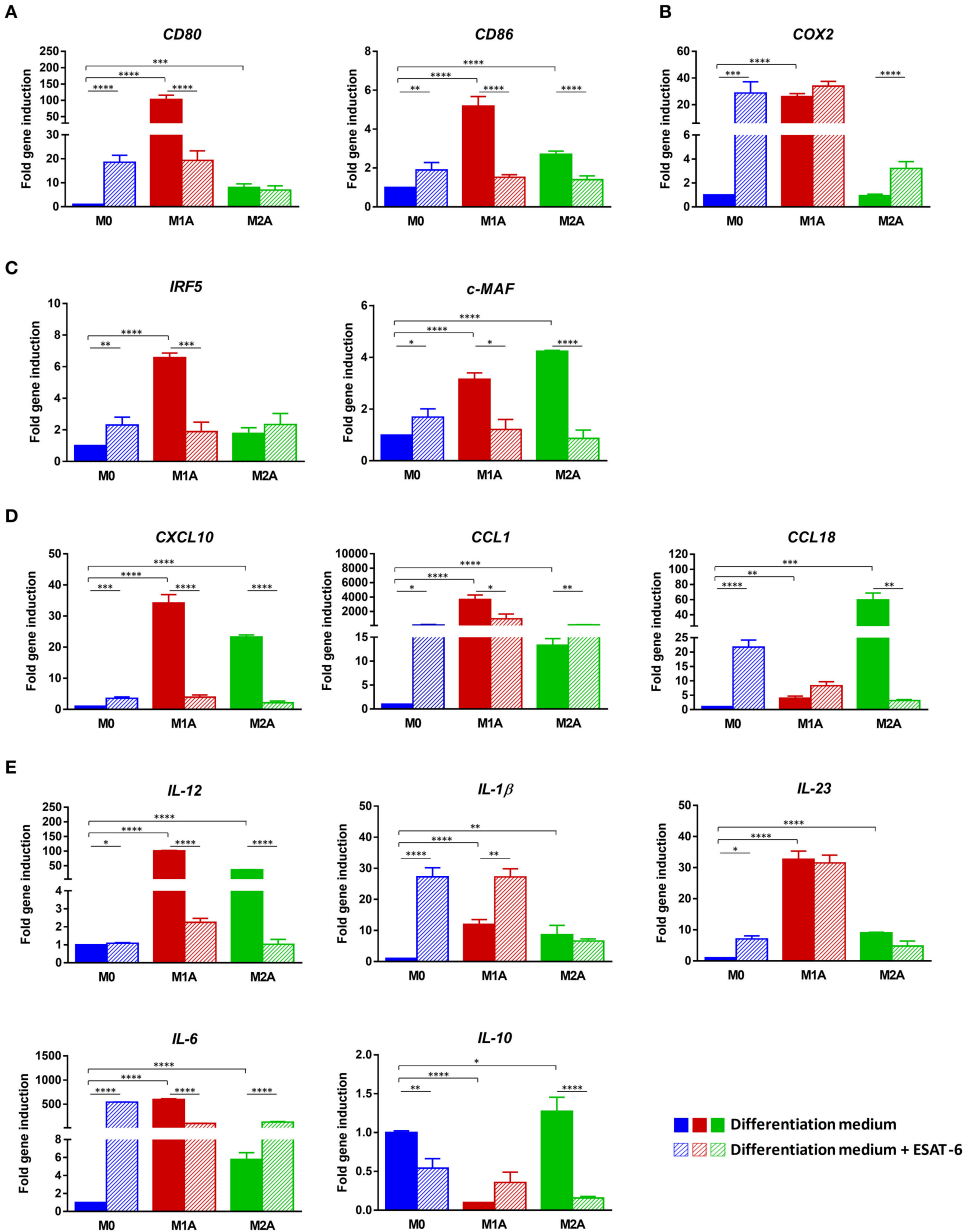
ESAT-6 modulates expression of M1A macrophage markers. RNAs were extracted from the MΦs as indicated in Figure 4 24 h after full activation in presence or absence of ESAT-6. Gene expression levels of **(A)** Cell surface molecules *CD80* and *CD86*, **(B)** *COX2*, **(C)** Transcription factors *IRF5* and *c-MAF*, **(D)** Chemokines *CXCL10, CCL1* and *CCL18*, as well as **(E)** Cytokines *IL-12, IL-1*β, *IL-23, IL-6*, and *IL-10* were assessed by RT-qPCR. Data are presented as the mean ±*SD* of 3 separate experiments (i.e. 3 different donors), each one carried out in triplicate. Asterisks indicate statistical significance (*p ≤ 0.05, **p ≤ 0.01, ***p ≤ 0.001, ****p ≤ 0.0001).

In order to further characterize the observed modulation of M1A MΦ pro-inflammatory profile induced by ESAT-6, we used RT-qPCR to analyze the expression profile of several M1 and M2 markers. We first confirmed the success in full activation of M1 and M2 MΦ subpopulations. Indeed, addition of LPS and IFN-γ to M1 MΦs at day 6 significantly induced two M1 markers *CD80* and *CD86* (*p* < 0.0001, Figure 5A) when compared to M0 MΦs. Similarly, *COX2* was significantly induced in M1A MΦs (*p* < 0.0001, Figure 5B) when compared to M0 MΦs, while such induction was not found in M2A MΦs (Figure 5B). As shown in Figures 5C–E, all the remaining M1 markers were also significantly induced in M1A MΦs when compared to M0 MΦs, with *p* < 0.0001 for *IRF5, CXCL10, IL-12, IL-1*β, *IL-23*, and *IL-6*. Of note, induction levels of *COX2, CXCL10, IL-12, IL-1*β, *IL-23*, and *IL-6* expression were much higher in M1A MΦs than in M1 MΦs (Figures 5A,D,E), thus supporting our success regarding *in vitro* M1 MΦ full activation. We also confirmed full activation of M2-differentiated MΦs by clear induction of M2 markers *c-MAF, CCL1* and *CCL18* (*p* < 0.0001, Figures 5C,D) when compared to that seen in M0 MΦs.

Induction folds of these 3 markers were much higher in M2A MΦs than in M2 MΦs. The immunosuppressive IL-10 was also significantly induced in M2A MΦs when compared to M0 MΦs (*p* < 0.05, Figure 5E). Combination of ESAT-6 with LPS and INF-γ significantly inhibited M1 markers *CD80* and *CD86* in M1A MΦs (*p* < 0.0001, Figure 5A). A similar modulation of M1 marker *IRF5* was also observed when M1A MΦs were treated with ESAT-6 (*p* < 0.001, Figure 5C), along with inhibition of M1 marker *CXCL10* (*p* < 0.0001, Figure 5D). A significant decrease in *IL-12* and *IL-6* expression levels (*p* < 0.0001, Figure 5E) was also observed in M1A MΦs when treated with ESAT-6. Moreover, a significant modulation of expression of three M1 markers *CD86, CXCL10*, and *IL-12* (*p* < 0.0001) was also observed even in M2A MΦs when they were treated with ESAT-6. Such treatment in M2A MΦs has also induced expression of M2 marker *CCL1* (*p* < 0.001, Figure 5C). However, on either M0 or M2A MΦs we found mixed effects of ESAT-6 on the remaining markers (Figures 5A–E). These data are in agreement with the above-described cytokine profiles (Figures 2A–D; Figures 4A–D) and suggest that ESAT-6 modulates pro-inflammatory pathways in M1A MΦs and switches them to the immunomodulatory phenotype.

In all of the results above, statistical analyses were first performed using the unpaired Student's *t*-test for simple comparison and the ANOVA for multiple comparisons, followed by additional verifications using the one-tailed version of the Mann Whitney test, which is more suited for small-sized samples (Salkind, 2007; Morgan, 2017). No substantial differences were found when comparing data obtained using either of the two statistical methods and, only *p*-values obtained using the former statistical tests were shown.

## Discussion

Alveolar MΦs, the first immunological barrier that opposes mycobacteria, are the primary conduit of tuberculosis infection and disease. In addition to recognition and immediate elimination of bacteria by phagocytosis and secretion of microbicidal products, MΦs are also extremely important in orchestrating the immune response and establishing a specific response provided by T cells. During its coexistence with humans, M.tb has developed several means to divert MΦ responses and has been shown to modulate effective immunity toward a tolerant steady state. Chiefs among these strategies are prevention of MΦ activation and establishment of an immunoregulatory M2-oriented phenotype (Sica et al., 2015). However, little is known about M.tb effectors that may interfere with MΦ differentiation and/or polarization.

Here, we investigated the role of ESAT-6 on these processes. We found that ESAT-6 drives differentiation of non-polarized M0 MΦs and anti-inflammatory M2 MΦs toward a pro-inflammatory M1 phenotype and subsequently induces the switch of fully active MΦs from the M1 to the M2 phenotype.

Data defining M1/M2 polarization are largely based on *in vitro* studies using monocytes isolated from peripheral blood. Thus, we used an *in vitro* system to generate different MΦ subpopulations (Figure 1) according to the recommended experimental protocols and guidelines (Murray et al., 2014). These experimental procedures are also in accordance with the fact that alveolar MΦs, the major players in immunity against tuberculosis, originate from peripheral monocytes (van Oud Alblas and van Furth, 1979). In M0 and M2 MΦs, addition of ESAT-6 to differentiation medium induced a typical M1 cytokine profile (Figure 2) and gene expression signature (Figure 3), while ESAT-6 did not interfere with M1 differentiation (Figures 2, 3). These observations indicate that ESAT-6 drives the differentiation of monocytes to a pro-inflammatory M1 MΦ profile, in agreement with a previous study indicating the potent activation of NLRP3/ASC inflammasome by ESAT-6 during *M.tb* infection (Mishra et al., 2010). Furthermore, it has also been reported that BCG complemented with ESAT-6 triggers an activation/inflammation program comparable to that induced by *M.tb* at an early stage of the infection, while parental BCG does not (Majlessi et al., 2005). Previous reports also indicated that M1 program is part of a common host immune response against intracellular bacteria (Ehrt et al., 2001; Deretic et al., 2004; Cairo et al., 2011). That is why we cannot exclude the presence of other *M.tb* effectors that may synergize with ESAT-6 to induce such primary pro-inflammatory M1 program.

Several studies carried out in humans and animal models showed that at an early stage of the infection, the anti-*M.tb* immune response is marked by an M1 polarization phenotype (Benoit et al., 2008; Lugo-Villarino et al., 2011). It is noteworthy that M1-type inflammatory signals induce more MΦ recruitment and lead to the formation of the primary/innate granuloma (Huang et al., 2015); a mechanism associated with MΦ apoptosis and, which is ESAT-6-dependent but T cell-independent (Davis and Ramakrishnan, 2009). Our data (Figures 2,3) with the fact that ESAT-6 is secreted early during the first phase of *M.tb* infection (Andersen et al., 1995; Sørensen et al., 1995) suggest that ESAT-6 contributes to the formation of the primary innate granuloma through induction of an M1-type MΦ differentiation program. Such local type 1 pro-inflammatory environment has been shown to overlap with that of IFN-γ (Ehrt et al., 2001) and would enhance more efficiently the recruitment of innate and adaptive immune cells, creating a bactericidal granuloma surrounded by Th1-type cells (Lugo-Villarino et al., 2013). However, it seems contradictory to assume that *M.tb* would secrete such a factor (ESAT-6) leading to its own destruction. Moreover, 90% of infected individuals do not succeed in eradicating the infection and remain latently infected bearing in their lungs “silent” solid granulomas containing slow-growing mycobacteria inside a particular MΦ subpopulation; FMs (Lugo-Villarino et al., 2013). Indeed, at a later stage of the infection, *M.tb* is shaping the granuloma function to its advantage and at the expense of the host via modulation of the M1 MΦ pro-inflammatory phenotype and polarization of these M1 MΦs toward an immunomodulatory M2 phenotype (Redente et al., 2010; Pessanha et al., 2012; Huang et al., 2015). When we combined ESAT-6 with LPS and IFN-γ at day 6 (M1 MΦ full activation cocktail, Figure 1B), we found a clear inhibition of the pro-inflammatory cytokine secretion and a significant induction of the immunosuppressive IL-10 (Figure 4). Expression levels of most of the studied M1A markers were also inhibited (Figure 5).

Importantly, we found that gene expression profile of *IL-1*β, *IL-23*, and *IL-10* did not corroborate these effects of ESAT-6 on fully activated M1 MΦs (Figure 5E). This could be explained by the low stability of the corresponding mRNAs that were remotely analyzed. Furthermore, ESAT-6 was also found to induce IL-6 secretion by M1A MΦs instead of its predicted inhibition (Figure 4B). IL-6 roles in tuberculosis are still controversial. Indeed, IL-6 has also been shown to inhibit type I interferon production by MΦs and modulate their responses to IFN-γ (Nagabhushanam et al., 2003). This may explain induction of IL-6 and IL-10 secretion by ESAT-6 and suggests that IL-6 production would enhance ESAT-6 anti-inflammatory effects. These data combined with our results (Figures 4, 5) suggest that ESAT-6 modulates pro-inflammatory pathways in M1A MΦs and switches the M1A phenotype to the immunomodulatory M2 phenotype. ESAT-6 has been reported to bind to TLR2, inhibiting the LPS-induced inflammatory response in murine MΦs (Pathak et al., 2007). This suggests TLR2 as one potential receptor through which ESAT-6 may have exerted its anti-inflammatory effects on M1A MΦs (Pathak et al., 2007). Further studies are needed to address the question of ESAT-6 and TLR2 relationships in tuberculosis and better characterize the cellular targets through which ESAT-6 inhibited M1A MΦ pro-inflammatory phenotype. It has also been reported that the M2 phenotype represents a transitional state preceding the formation of FMs; cells known to be rich in lipid bodies and the carbon source of dormant intracellular mycobacteria (Peyron et al., 2008; Russell et al., 2009; Griffin et al., 2011). Moreover, ESAT-6 has been identified as the molecular mediator used by *M.tb* to induce metabolic flux perturbations leading to differentiation of FMs (Singh et al., 2012, 2015). These data strongly support our findings and suggest ESAT-6 as being a *M.tb* effector driving polarization of MΦs toward the M2 immunomodulatory phenotype and inducing FM differentiation at a late stage of the infection. These two major cells would contribute to the formation of a “silent” solid granuloma with the predominance of Th2 and Treg immune responses (Lugo-Villarino et al., 2011).

We are aware that working with a limited number of human samples (i.e., 3) may not guarantee a normal statistical distribution, thus more volunteers (*n* > 7) should be included in future studies in order to strengthen our data.

**Figure 6:**
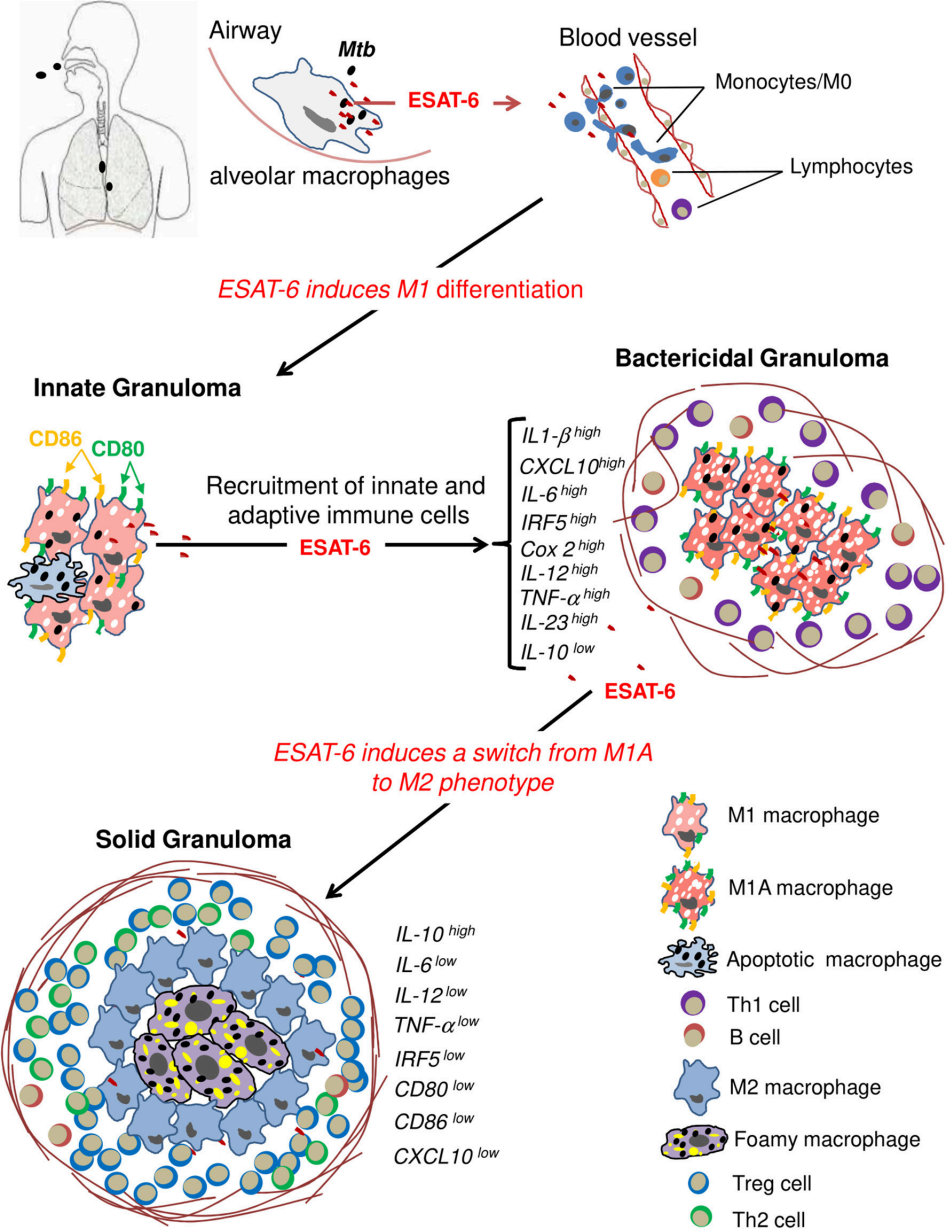
ESAT-6 drives host macrophage differentiation and activation as a mechanism to first induce granuloma formation and subvert the immune response at a later stage of the infection. Resident classical M1 alveolar MΦs are the first encounters of inhaled mycobacteria. The latter ones proliferate inside the phagosome with a concomitant secretion of ESAT-6, which disseminates to nearby blood vessels and contributes to differentiation of peripheral monocytes into M1-type MΦs. The local type-1 pro-inflammatory environment along with apoptotic MΦs would further enhance recruitment of innate and adaptive immune cells, creating a bactericidal granuloma. In order to shape granuloma function to its advantage, *M.tb* secretes ESAT-6, which modulates MΦ pro-inflammatory M1 phenotype and induces polarization of M1 MΦs toward the immunomodulatory M2 phenotype. Part of these cells will differentiate into FMs under the action of ESAT-6. FMs represent the ideal niche for *M.tb* to survive in and proliferate slowly. M2 MΦs and FMs contribute to the formation of a “silent” solid granuloma with predominance of Th2 and Treg immune responses.

Collectively, our results suggest that ESAT-6 acts as a key virulent effector used by *M.tb* to contribute to host MΦ differentiation and activation as a mechanism to first induce granuloma formation and later subvert the immune response in order to maintain a persistent infection (Figure 6).

Our study also provides new insights into roles of ESAT-6 in the pathophysiology of *M.tb* infection and may be helpful for the development of novel and more efficient drugs that target ESAT-6 for a better treatment and/or prevention of tuberculosis.

## Author contributions

ME and AR conceived, designed and performed the experiments. AR, SG, and ME analyzed the data and interpreted them and wrote the paper as well. M-RB contributed with providing reagents and discussing the manuscript.

### Conflict of interest statement

The authors declare that the research was conducted in the absence of any commercial or financial relationships that could be construed as a potential conflict of interest.
